# Synergetic Effects of* Plasmodium*, Hookworm, and* Schistosoma mansoni* Infections on Hemoglobin Level among Febrile School Age Children in Jawe Worda, Northwest Ethiopia

**DOI:** 10.1155/2018/9573413

**Published:** 2018-06-12

**Authors:** Tadesse Hailu, Mulat Yimer, Wondemagegn Mulu, Bayeh Abera

**Affiliations:** Department of Microbiology, Immunology and Parasitology, College of Medicine and Health Science, Bahir Dar University, Bahir Dar, Ethiopia

## Abstract

Plasmodium coinfection with hookworm and/or* Schistosoma mansoni* has detrimental effects on human's hemoglobin level. This study aimed to determine the effects of plasmodium, hookworm, and* S. mansoni* infections on hemoglobin level among febrile school age children in Northwest Ethiopia. A cross-sectional study was conducted from April 2016 to August 2016.* Plasmodium* and helminths infections were detected using Giemsa stain and formol-ether concentration techniques, respectively. Hemoglobin level was determined using Hemocue method. Among 333 children, 143 (42.9%), 49 (14.75%), and 22 (6.6%) had* Plasmodium, hookworm, and Schistosoma mansoni* infections, respectively. The prevalence of* Plasmodium*-hookworm and* Plasmodium*-*Schistosoma mansoni* coinfections was 18 (12.6%) and 4 (2.8%) in children, respectively. The overall prevalence of anaemia in children was 41.4%. Effect of* Plasmodium*, hookworm, and* Schistosoma mansoni* on hemoglobin level was high. Therefore, febrile children should be screened for* Plasmodium*, hookworm,* Schistosoma mansoni,* and anaemia simultaneously in malaria endemic areas.

## 1. Introduction

Plasmodium, hookworm, and* Schistosoma (S) mansoni *infections are the most important public health problems that cause devastating effect among children especially in the developing world [1]. Approximately, there were an estimated 216 million cases and 445 000 deaths of malaria worldwide [2]. Of these mortalities, 90% occur in African children especially in Sub-Saharan Africa [3]. More than 1 billion people become infected, and 450 million are ill due to helminths parasite infections [4].

Infection of* Plasmodium falciparum* develops severe malaria manifested by severe anaemia complication. Children are at high risk population groups to develop severe anaemia in areas where prevalence of* P. falciparum* is high [5]. Similarly, infections with hookworm and* S. mansoni* lead to malnutrition, iron deficiency anaemia, fetal stunted growth, increased vulnerability to other infections [6], and low educational achievement in school children [7].

In areas 2000 m below sea level,* Plasmodium*, hookworm, and* S. mansoni* infections are coendemic [8]. Children who live in endemic areas of the above parasites are vulnerable to be infected by these parasites.* Plasmodium,* hookworm, and* S. mansoni* affect the red blood cells and causes anaemia (hemoglobin level <11 g/dl) especially in children [9]. Effects of malaria, hookworm, and* S. mansoni* cause severe anaemia and have considerable impact on children health. Children having anaemia as well as other parasitic infections are a number of times more likely to be stunted and underweight than those who do not [10]. Institution based information indicated that the prevalence of malaria is the primary disease among the ten top diseases and hookworm and* S. mansoni* infections are also high in Jawe Woreda. However, the available information on the effect of* Plasmodium*, hookworm, and* S. mansoni* on hemoglobin level in Ethiopia in general and in the study area in particular is lacking. Therefore, this study aimed to determine the effects of plasmodium, hookworm, and* S. mansoni* infections on hemoglobin level among febrile school age children in Northwest Ethiopia.

## 2. Material and Methods

### 2.1. Study Design, Period, and Area

A cross-sectional study was conducted from April 2016 to August 2016 among febrile school age children in Jawe Woreda, Awe Zone, Amhara regional state, Northwest Ethiopia. The altitude of the Woreda is between 648 and 1300 meters. The annual temperature of the study area ranges between 16.68°C and 37.6°C. The average annual rain fall is 1569.4 mm. The Jawe Woreda is a potential irrigation area using Tana-Beles water source. This water harvesting might be potential for preexistence of* Plasmodium* and* S. masoni *infections. A total of 333 febrile school age children were included in this study. Systematic random sampling technique was conducted until the required sample size is achieved. The samples were collected in Jawe Health Center and Workmeda Health Center. The sample size in each health center was allocated by considering the population in the catchment areas.

All febrile children age ranging from 6 to 14 years, attending Jawe Health Center and Workmeda Health Center, and willing to participate in the study were included in the study. Febrile children who had fever >37°C were included in the study. Children taking antimalaria or/and antihelminthic drugs during the data collection time were excluded.

### 2.2. Data Collection

Demographic information, explanatory variables, and environmental related factors were collected via face to face interview of parents/guardian of the children. All information was filled on a structured questionnaire by health officers.

### 2.3. Stool Sample Collection

Freshly passed stool specimens were collected using clean plastic cup at the two health centers.

To detect diagnostic stages of helminths in direct microscopy, approximately, 20 mgs of fresh stool sample was put on a slide with wooden applicator, emulsified with a drop of physiological saline (0.85 %), covered with cover slide, and examined under microscope using first 10X objectives and then 40X objectives.

In FECT, 0.5 g of fresh stool sample was transferred into a test tube which contains 10 mL of normal saline and mixed thoroughly. Two layers of gauze were placed in a funnel. Well mixed stool sample is strained into a 15 mL centrifuge tube. Then 2.5 mL of 10 % formaldehyde and 1 mL of ether were added to the test tube which contains the sample. The test tubes were mixed well until homogenized and centrifuged at 1000 revolution for three minutes. The supernatant was discarded and the sediment was mixed well and a drop of the sediment was put on slide and covered with cover slide and saw first with 10X and then 40X objective microscope.

### 2.4. Blood Sample Collection, Preparation, and Examination

Blood samples were collected by finger prick from each study participant for parasite detection and hemoglobin determination. Thick and thin blood films were prepared on a single slide. In the thick film preparation, three drops of blood were distributed over an area of 1 cm^2^ at one end of the slide. Thin smear was also done by evenly distributing a drop of blood on the other end of the slide. Slides were labeled, dried, fixed with methanol alcohol (thin smear only), stained (using 3 % Giemsa stain solution for 30 minutes), washed with distill water, and air dried.* Plasmodium *was detected on the thick blood films, whereas species identification was done on the thin film.

Hemoglobin was determined by Hemocue method using Hemocue Hb 201 analyzer (HemoCue, Angelholm, Sweden) and specially designed microcuvette (the Hemocue Hb 201 Microcuvette, Hemocue, Angelholm, Sweden). Blood was taken from finger prick using microcuvette and inserted to the analyzer until the analyzer displays the result. Hemoglobin level below 11 g/dL for children indicates anaemia [9].

### 2.5. Quality Control

To ensure reliable data collection, training of laboratory technicians and health officers on data collection and explanation about the study was given before sample collection. Application of standard procedures and accuracy of test results were supervised by principal investigator. The stool cups were labeled based on their serial number. The direct stool microscopy was examined earlier to FECT as soon as the sample arrived. To eliminate observer bias, thick blood films and FECT stool slides were examined independently with two experienced laboratory technicians and 10 % of the thick blood film and FECT slides was randomly selected and read by another technician as a quality control. The results of their observation will be recorded for later comparison on separate sheets.

### 2.6. Data Analysis

Data was entered and analyzed using SPSS version 20 statistical software. Overall magnitude and coinfection of* Plasmodium* with hookworm and* S. mansoni *and hemoglobin level were calculated using descriptive statistics and chi-square. The differences were considered to be statistically significant if* p-value* was < 0.05.

## 3. Result

### 3.1. Demographic Characteristics of Study Participants

A total of 333 febrile school age children were included in this particular study. The range and mean age of the study participants' were 8 and 11 years, respectively. The majority of cases (74.2 %) were primary and junior school students. Most of the participants (98.5 %) were Christian. Two hundred seventy-three (82 %) febrile cases were rural inhabitant. Jawe Health Center accounted for 52.3 % of the participants (Table 1).

### 3.2. Plasmodium, Hookworm, and S. mansoni Infections

The prevalence of plasmodium infection among febrile school age children was 143 (42.9 %), of which,* P. falciparum *infection accounted for 137 (97.2 %) among the* Plasmodium* infected cases. The prevalence of plasmodium infection in rural residents was 116 (42.5 %). The prevalence of* P. falciparum *accounted for 95.8 % among malaria infected febrile children (Table 2).

The prevalence of hookworm and* S. mansoni* infections was 49 (14.7 %) and 22 (6.6 %), respectively. Ten (3.6 %) of children developed double infection with hookworm and* S. mansoni* (Table 2). There were also 141 (42.3 %) children infected with single parasite.

### 3.3. Plasmodium-Helminths Coinfection

The prevalence of* Plasmodium*-hookworm and* Plasmodium*-*S. mansoni* coinfection among febrile children was 12.6 % and 2.8 %, respectively. The prevalence of triple infection with* Plasmodium*-hookworm-*S. mansoni* among plasmodium infected children was 4.2 % (Table 3).

### 3.4. Hemoglobin Determination

The overall prevalence of anaemia was 41.4 %. The prevalence anaemia among noninfected and coinfected children was 35.8 % and 85.3 %, respectively (Table 4). The range and mean hemoglobin level was 7g/dL and 10.9g/dL, respectively. The prevalence of anaemia among single parasite infected children was 19.5 %.

The mean hemoglobin level noninfected children were 11.33 g/dL. Low mean was recorded in hookworm infected children (9.84 g/dL) among single parasite infected children*. S. mansoni-P. falciparum* coinfected children had low hemoglobin level (9 g/dL). Triple infected children had 8.67 g/dL mean hemoglobin level (Table 5).

## 4. Discussion

This study may be considered the first study conducted on the effects of* Plasmodium*, hookworm, and* S. mansoni* infections on hemoglobin level in Jawe Woreda. These parasites have high prevalence in areas less 2000 m below sea level [8, 11]. The presence of the above parasites in the same geographical areas will have a devastating effect in the health of children since they cause anaemia.

The overall prevalence of* Plasmodium* (42.9 %) infection was high and* P. falciparum* was the predominant species in this study. This finding was lower than a study done in Southern Ethiopia (82.84%) [12] and Zambia (50.6 %) [1], but higher than a study done in Malawi (31 %) [13]. This difference may be due to difference in data collection time, altitude, and geographical factors. In the presence study, we collected the sample during low transmission season.

Hookworm (14.7 %) was the most prevalent helminths infection in the present study which was similar to the previous study done in Southern Ethiopia (37.8 %) [14]. Lower prevalence of hookworm among school age children in this study was recorded when compared to the previous study done in Southern Ethiopia (26.8 %) [15] and Zambia (42.4 %) [1]. This difference might be due to difference in wearing of shoe and environmental factors like soil type.

The distribution of* S. mansoni* (6.6 %) in the present study was low than previous study done in Southwest Ethiopia (11.7 %) [16] and Northwest Ethiopia (49 %) [17]. This might be due to the method difference that we used to diagnoses* S. mansoni.* Kato-Katz technique is the gold standard method for the diagnoses of* S. mansoni.*

Prevalence of coinfection of* Plasmodium*-hookworm (12.6 %) and* Plasmodium*-*S. mansoni *(2.8 %) in the present study was comparable with a previous study done in Zambizi (11.8 %) and (2.7%), respectively [1], but triple infection (4.2 %) in the current study was higher than triple infection (2.7 %) result obtained in Zambia [1]. Prevalence (100 %) in triple infection was found only in Malaria-hookworm-*S. mansoni* parasites. This might be due to the endemicity and geographical location or altitude <2000 m below sea level where these parasites are coendemic.

The prevalence of* Plasmodium*-*S. mansoni* coinfection found in this study caused high prevalence of anaemia as compared to those infected only with malaria in the present study. This was comparable with previous study done in Southern Ethiopia which showed that* Plasmodium*-*S. mansoni *infected children were 1.6 times more likely to be anemic compared to those with Plasmodium infection alone [16].

The prevalence of* Plasmodium*-*S. mansoni *(2.8 %) in the present study was comparable with a study conducted in Kinshasa (1.5 %) [18] but lower than previous study done in Southwest Ethiopia (38.5 %) [16] and Northwest Ethiopia (19.5%) [19]. The current low prevalence may be due to method deference.

The prevalence of anaemia (41.4 %) among school age children in the presence study was comparable with previous study done in Kinshasa (41.6%) [18] but higher than a study done in Southeast Ethiopia (23.66 %) [20] and Southwest Ethiopia (37.6 %) [21]. This finding was also lower than a study conducted in Southern Ethiopia (20.6 % ) [15] and Yemen (46.0%) [22] and Egypt (59.3%) [23]. Level of hemoglobin concentration infected with helminthes and* Plasmodium* in the presence study was lower. This finding was comparable with other findings in Ethiopia [14].

## 5. Conclusion

Malaria, hookworm, and* S. mansoni* are coendemic and their prevalence is high in school age children. Coinfected (*Plasmodium*-hookworm and malaria-*S. mansoni*), double infected (hookworm and* S. mansoni),* and triple infected (*Plasmodium*-hookworm-*S. mansoni* ) children have low levels of hemoglobin. Therefore, febrile cases should be diagnoses for* Plasmodium* and helminths infection simultaneously in malaria endemic areas.

## Figures and Tables

**Table 1:** Demographic characteristics of school age children in Northwest Ethiopia, 2016.

**Variables**		**Distribution **		
		**Workmeda Health Center (N, %)**	**Jawe Health Center** **(N, %)**	**Total** **(N, %)**
**Age**	**6-8**	**26 (36.6)**	**45 (63.4)**	**71 (21.3)**
	**9-11**	**36 (40.4)**	**53 (59.6)**	**89 (26.7)**
	**12-14**	**97 (56.1)**	**76 (43.9)**	**173 (52)**
**Sex**	**Male**	**68 (41.7)**	**95 (58.3)**	**163 (48.9)**
	**Female**	**91 (53.5)**	**79 (46.5)**	**170 (51.1)**
**Religion**	**Christian**	**159 (48.5)**	**169 (51.5)**	**328 (98.5)**
	**Muslim**	**0 (0.0)**	**5 (100)**	**5 (1.5)**
**Residence**	**Rural**	**134 (49.1)**	**139 (50.9)**	**273 (82)**
	**Urban**	**25 (41.7)**	**35 (58.3)**	**60 (18)**
**Educational status**	**Illiterate**	**79 (91.9)**	**7 (8.1)**	**86 (25.8)**
	**Primary (1-4)**	**50 (30.1)**	**116 (69.9)**	**166(49.9)**
	**Junior (5-8)**	**30 (37)**	**51 (63)**	**81 (26.3)**
**Total**		**159 (47.7)**	**174 (52.3)**	**333 (100)**

**Table 2:** Distribution of plasmodium and helminths infection among school age children in Northwest Ethiopia, 2016.

**Types of parasites**		**Age category**				** P-value**
		**6-8** **(N, %)**	**9-11** **(N, %)**	**12-14** **(N, %)**	**Total** **(N, %)**	
**Plasmodium**	**Negative**	37 (19.4)	49 (25.9)	104 (54.7)	190 (57.1)	
	**PF**	34 (24.8)	39 (28.5)	64 (46.7)	137 (41.1)	**0.83**
	**PV**	0 (0.0)	1 (16.7)	5 (83.3)	6 (1.8)	
**Helminths**	**Negative**	62 (22.6)	76 (27.8)	136 (49.6)	274 (82.3)	
	**HW**	7 (18.9)	7 (18.9)	23 (62.2)	37 (11.1)	
	**SM**	0 (0.0)	4 (40)	6 (60)	10 (3.0)	
	**SM + HW**	2 (16.7)	2 (16.7)	8 (66.6)	12 (3.6)	
**Total**		71 (21.3)	89 (26.7)	173 (52)	333 (100)	0.34

HW: Hookworm, SM: *S. mansoni*, PF: *P. falciparum*, and PV: *P. vivax.*

**Table 3:** Distribution of helminths in plasmodium infected school age children in Northwest Ethiopia, 2016.

***Plasmodium***	**HW**	**SM**	**HW + SM**	**Neg**	**Total**	***P*-value**
**PF**	**15 (11)**	**3 (2.2)**	**6 (4.4)**	**112 (82.4)**	**136 (95.1)**	
**PV**	**3 (42.9)**	**1 (14.2)**	**0 (0.0)**	**3 (42.9)**	**7 (4.9)**	
**Total**	**18 (12.6)**	**4 (2.8)**	**6 (4.2)**	**115 (80.4)**	**143 (42.9)**	**0.02**

HW: Hookworm, SM: *S. mansoni,* PF: *Plasmodium falciparum*, PV: *Plasmodium vivax*, and Neg: negative.

**Table 4:** The prevalence of anaemia among coinfected children in Northwest Ethiopia, 2016.

	**Negative (N, %)**	**Co-infection (N, %)**					**Total**
**HB** **level **		**P-HW**	**P-SM**	**HW-** **SM**	**P-HW-** **SM**	**Total**	
**<11g/dL**	107 (35.8)	16 (88.9)	3 (66.7)	5 (83.3)	5 (83.3)	29 (85.3)	136 (40.8)
**≥11g/dL**	192 (64.2)	2 (11.1)	1 (33.3)	1(16.7)	1 (16.7)	5 (14.7)	195 (59.2)
**Total**	299 (89.8)	18 (5.4)	4 (1.2)	6 (1.8)	6 (1.8)	34 (10.2)	333
***P-value***		<0.00	<0.1	<0.00	<0.00	<0.00	<0.00

HB: hemoglobin, P: *Plasmodium*, HW: hookworm, and SM: *S. mansoni.*

**Table 5:** The mean values of hemoglobin among noninfected and infected children in Northwest Ethiopia, 2016.

**Variables**	**N**	**Mean Hemoglobin (g/dL)**	**95 % CI**
**Non Infected **	159	11.33	9.90 – 12.77
**Single Infection**			
PF	112	10.79	10.53 – 11.04
HW	19	9.84	9.51 – 10.17
SM	6	11.33	10.25 – 12.42
**Double Infection**			
PF + SM	3	9	6.52 - 11.48
PF + HW	15	9.20	8.72 – 9.68
SM + HW	6	9.67	9.12 – 10.21
**Triple infection**			
PF + SM + HW	6	8.67	7.23 – 10.10

PF: *P. falciparum*, HW: hookworm, and SM: *S. mansoni.*
